# Ratio of Nitric Oxide Metabolite Levels in Cerebrospinal Fluid and Serum, and Their Correlation with Severity and Outcome in Patients with Subarachnoid Haemorrhage

**DOI:** 10.21315/mjms2021.28.6.5

**Published:** 2021-12-22

**Authors:** Giat Seng KHO, Regunath KANDASAMY, Mohamad Adam BUJANG, Mummedy SWAMMY, Muzaimi MUSTAPHA, Jafri Malin ABDULLAH

**Affiliations:** 1 Department of Neurosciences, School of Medical Sciences, Universiti Sains Malaysia, Kubang Kerian, Kelantan, Malaysia; 2 Department of Neurosurgery, Sarawak General Hospital, Kuching, Sarawak, Malaysia; 3 Clinical Research Centre, Sarawak General Hospital, Ministry of Health Malaysia, Sarawak, Malaysia; 4 Department of Chemical Pathology, School of Medical Sciences, Universiti Sains Malaysia, Kubang Kerian, Kelantan, Malaysia

**Keywords:** aneurysm, subarachnoid haemorrhage, nitric oxide metabolites, CSF-to-serum ratio, outcome

## Abstract

**Background:**

Nitric oxide (NO) is involved in a multitude of physiological processes in the central nervous system (CNS). Given the ubiquitous nature of NO and its involvement in various vital processes, nitric oxide metabolite (NOx) has been investigated as a biomarker in CNS diseases. This study aims to investigate the ratio of NOx levels and serum in cerebrospinal fluid (CSF) in patients with spontaneous subarachnoid haemorrhage (SAH). The associations among these markers with clinical outcomes were also studied.

**Methods:**

A prospective cohort study was conducted over a 2-year period (May 2013–May 2015) to investigate the levels of NOx in the CSF and serum of patients with radiologically confirmed aneurysmal SAH. NOx samples and all relevant data were collected from the patients on admission and serially over 5 days. On admission, NOx levels were compared between the groups of patients, who were divided as per the World Federation Neurosurgeons Score (WFNS) grading scale, Fisher scale, occurrence of vasospasm on transcranial doppler (TCD), and Glasgow outcome scale (GOS) upon discharge and at 6 months follow-up. The ratios of CSF-to-serum were calculated and correlated with SAH severity and the outcome parameters listed above.

**Results:**

The patients (*N =* 40) had a mean (SD) age of 58.2 (11.8) years old. The majority (65%) had a higher severity of SAH (WFNS score 3–5). On evaluation of the CT scan findings, 74% had outcomes equivalent to 4 on the Fisher scale. Vasospasm was detected via TCD in nearly half (45%) of the cohort during the study period; 80% were noted to have a poor outcome (GOS 1–3) at discharge; this persisted at 6 months follow-up. Comparison of NOx levels in the CSF/serum ratio was based on the incidence of vasospasm and severity of outcome (GOS) for day-1 and day-4. Statistically significant results were evident for patients with better outcomes, high severity grading, and the presence of vasospasm (*P*-values: 0.031, 0.034 and 0.043, respectively).

**Conclusion:**

Elevated NOx levels in CSF and serum and reductions in the ratio of NOx in CSF/serum were found to be associated with severity, occurrence of vasospasm and clinical outcome in aneurysmal SAH patients. This indicates the possible role of NOx as a biomarker to assess severity and prognosis in patients with SAH.

## Introduction

Subarachnoid haemorrhage (SAH) describes the extravasation of blood between the pia and arachnoid layers of the brain. SAH occurring spontaneously in the absence of trauma is well recognised as being due to the rupture of an intracranial aneurysm and accounts for 6%–8% of all strokes (1). Aneurysmal SAH is associated with high morbidity and mortality, mainly due to the effects of the initial haemorrhage, re-bleeding and delayed ischaemic neurological deficits from vasospasm (2). Management of patients with SAH usually entails admission to a neurocritical care unit followed by surgery or endovascular therapy to occlude the aneurysm and perform concurrent aggressive therapeutic measures to counter any secondary processes that might complicate the patient’s recovery (1).

Patient outcomes in SAH remain difficult to predict; the clinical course of disease differs substantially due to initial clinical status, aneurysm size and underlying comorbidities, among other factors (3). The World Federation Neurosurgeons Score (WFNS) grading scale is one of the commonly utilised clinical grading systems for prognosticating survival in patients with SAH (4). The WFNS grading scale is objectively based on the Glasgow coma scale score on admission and the presence of neurological deficits. Other commonly used grading systems include the Hunt and Hess grading scale and the Fisher grading scale (5–6). Despite the existence of these systems, accurately predicting clinical outcomes remains difficult; often a clinician needs to rely on experience to guide their therapeutic decision-making. Efforts have been made in recent times to create predictive models that combine patient and disease characteristics. One noteworthy system created by Jaja et al. (7) is based on data from over 5,000 patients with SAH. The model highlights that age, WFNS score, volume of SAH, and size and location of the aneurysm all significantly influence clinical outcomes. The validity of this system, however, remains under investigation: criticisms include its biases in patient selection and reliability, confounded by measurement errors of the listed parameters.

Outcome biomarkers are parameters that can be objectively measured or evaluated as indicators of the outcome of a disease or pathological process. In the central nervous system (CNS), nitric oxide (NO) is involved in a multitude of physiological processes including the maintenance of basal vasomotor tone, inhibition of platelet and leukocyte aggregation, macrophage-mediated cytotoxicity, neurotransmission, selective neuroprotection, apoptosis, synaptogenesis, intercellular signalling, synaptic plasticity and memory formation. In pathological states, both excesses and deficiencies of NO may exist and have deleterious effects like hypoperfusion and direct neurotoxicity (4–5). The ubiquitous nature of NO and its involvement in various vital processes have resulted in NO being investigated as a biomarker in CNS diseases (8–10). In humans, while direct measurement of NO is impractical, the end products of NO metabolism, NOx, can be studied as an index of NO production (8).

Several clinical studies represent attempts by various authors to determine the role of NO as a potential biomarker of severity and outcome related to SAH (8, 11–12). The vast amount of data concurs on the principal effects of NO on patients following SAH. However, no agreement exists on the exact conditional interactions among the different mechanisms. Thus, further studies are required to determine the nature of these relationships and to investigate the role of NO as a biomarker in SAH. With this in mind, the current study aimed to measure NOx levels in cerebrospinal fluid (CSF) obtained via ventriculostomy in patients admitted with SAH and to correlate the levels with clinical and radiological grading systems and transcranial doppler (TCD) velocities (13).

Additionally, we aimed to measure the serum levels of NOx to calculate the ratio between NOx levels in CSF and serum. Previous authors have noted that CSF NOx levels correlated with serum NOx levels (11, 14). The exact mechanism by which this occurs has not been unequivocally proven. It is known that elevated CSF NOx levels in patients occur due to the enhanced production of NOx by an injured brain (15). It is also thought that elevated NOx levels within serum occurs due to leakage from the CNS caused by impairment in the blood-brain barrier (BBB) that occurs following SAH (16). Based on this premise, elevated levels of NOx in serum should directly correlate with the severity and outcome of SAH (15). Contrastingly, studies of serum NOx levels have thus far failed to reveal a significant correlation with outcome or severity (2, 17).

It has been hypothesised that a more severe incidence of SAH results in a greater degree of BBB dysfunction (18). This further translates into an increased amount of NOx leaking from the CNS into the serum. The systemic complications of SAH may also contribute to increased NOx levels in the serum (19). Due to the various confounding factors that may influence the absolute value of serum NOx levels, we hypothesised that changes in the ratio of CSF-to-serum NOx levels may better correlate with severity and outcomes following SAH rather than absolute values. In this study, we determined the ratio of CSF-to-serum NOx levels and determined the values that significantly predict outcomes in this group of patients.

The main objective was to study NOx levels in CSF and serum in patients with SAH. To achieve this, we compared (i) the ratio of NOx levels in CSF and serum between patients with lower and high severity SAH based on the WFNS grade on admission and CT scan findings of patients classified according to the Fisher scale for SAH; (ii) the mean values and ratio of NOx levels in CSF and serum between patients with and without vasospasm as measured via mean TCD velocities of the middle cerebral artery; (iii) the mean values and ratios of NOx levels in CSF and serum between patients with poor and good outcome based on the GOS of patients at 6 months post-ictus; and (4) the temporal changes in mean values and the ratio of CSF and serum NOx metabolites via sampling throughout the duration requiring CSF diversion.

## Methods

This prospective cohort study was conducted at the Hospital Universiti Sains Malaysia (HUSM), Kubang Kerian, Malaysia (May 2013–May 2015). CSF samples, serum samples, and all relevant data were collected from the patients on admission and serially over 5 days. The sampling frame consisted of all adult patients aged 18 years old and above admitted to HUSM with SAH diagnosed based on a CT brain scan taken on admission. The aneurysm was diagnosed and located using CT angiography and/or digital subtraction angiography (DSA) of four blood vessels in the brain.

This study aimed to describe and compare the NOx levels of CSF and serum in patients categorised by clinical and radiological severity, occurrence of vasospasm and outcome. The parameters measured included WFNS grading scale on admission, CT Fisher scale, TCD values, and GOS upon discharge and at 6 months follow-up. The trends in CSF and serum NOx over a 5-day period were used to determine any correlations among these values with the above-listed predictors of severity and outcome in accordance with the day on which the sampling was performed.

CSF NOx was measured as nitrate/nitrite using the nitrate/nitrite colourimetric assay kit (Cayman Chemical Company, Ann Arbor, MI, USA). This kit utilises a two-step process for the accurate and convenient measurement of total nitrate/nitrite. The first step involves the conversion of nitrate to nitrite using nitrate reductase; the second step involves the addition of the Griess reagents, which converts nitrite into a deep purple azo compound. Photometric measurement of the absorbance due to this azo chromophore accurately determines the nitrite concentration.

### Statistical Analysis

Data entry and analysis were performed using IBM SPSS (version 22) (IBM Corp. Released 2013. IBM SPSS Statistics for Windows, Version 22.0 Armonk, NY: IBM Corp.). Means and standard deviations were calculated for CSF and serum NOx based on severity grading, outcome and vasospasm. Independent sample *t*-tests were performed to compare mean CSF and serum NOx between the patient groups by severity and outcome. Pearson’s Chi-square test was used to determine the association between the ratio of CSF-to-serum NOx levels and the listed severity and outcome parameters. The significance level was set at a *P*-value of < 0.05 (2-sided).

## Results

### Descriptive Data

The patients (*N* = 40) were aged 30–77 years old with a mean age of 58.2 years old (SD 11.8) (Table 1). Males represented 30% of the sample population; the majority (92.5%) were of Malay ethnicity. The majority (65%) had a high severity of SAH (WFNS 3–5). On evaluation of the CT scan findings, 74% were categorised with a Fisher scale of 4. Vasospasm was detected via TCD in nearly half (45%) of the cohort during the study period. Eighty percent had a poor outcome (GOS 1–3) at discharge; this persisted at 6 months follow-up.

The overall mean NOx value in CSF, serum and CSF-to-serum ratio in the study population was 9.16 μmol/L (SD 2.25), 51.48 μmol/L (SD 14.69) and 0.364 (SD 0.13), respectively (Table 2). On review of the temporal changes from day-1 to day-5, CSF NOx levels ranged from 6.3 μmol/L–11.3 μmol/L while serum NOx levels ranged from 36.6 μm–68.7 μm. These values steadily decreased till day-4 before plateauing at day-5 (Tables 3 and 4). The CSF-to-serum ratio ranged from 0.281–0.592 and increased significantly over the 5-day study period (Table 5).

### Univariate Analysis

#### Comparison of NOx Levels in CSF and Serum from Day-1 to Day-5 between Groups of Patients Based on Severity Grading (WFNS)

The mean values of CSF NOx metabolites were significantly higher in patients with a higher severity grading on all days (*P*-value < 0.001). Serum NOx levels also demonstrated similar trends in those with severe SAH. Serum NOx levels were only significantly different between the groups on day-4 and day-5 of sampling (*P*-values: 0.005 and 0.003, respectively) (Table 3). The temporal profile showed an initial peak, followed by a subsequent decline in levels of both CSF and serum in severity grading (Tables 3 and 4). Comparison of CSF and serum NOx levels showed significant differences over time in patients with low severity on day-1 and day-4 (Table 6).

#### Comparison of NOx Levels in CSF and Serum from Day-1 to Day-5 between Groups of Patients Based on Occurrence of Vasospasm

The mean values of CSF NOx levels were significantly higher in patients with vasospasm on all days (*P*-value < 0.001). Serum NOx levels also demonstrated the same trends in those with vasospasm but were not statistically significant on all days (Tables 3 and 4). The temporal profile showed an initial peak, followed by a subsequent decline in serum NOx levels for both CSF and serum in those with vasospasm (Tables 3 and 4). A comparison of CSF NOx levels in patients with/without vasospasm on day-1 and day-4 was not significant (Table 6). A comparison of serum NOx levels in patients with an occurrence of vasospasm from day-1 to day-4 showed significant differences (*P*-values 0.042 and 0.020, respectively).

#### Comparison of NOx Levels in CSF and Serum from Day-1 to Day-5 between Groups of Patients Based on Outcome

Mean CSF NOx level values were significantly higher in patients with poor outcomes from day-3 to day-5 (*P*-values 0.03, 0.006 and 0.011, respectively). Serum NOx levels were significantly higher in patients with good outcomes on day-1 and day-2 (*P*-values 0.01 and 0.017, respectively) (Tables 3 and 4). On review, the temporal changes from day-1 to day-5, CSF and serum NOx levels in patients with poor and good outcomes also demonstrated the same trends as vasospasm and severity (Tables 3 and 4). A comparison of CSF and serum NOx levels in patients based on outcome from day-1 to day-4 showed only significant differences in patients with poor and good outcomes (*P*-values 0.013 and 0.05, respectively) (Table 6).

#### Comparing the Ratio of NOX in CSF/Serum from Day-1 to Day-5 between Patients Divided Based on Severity, Occurrence of Vasospasm and Outcome

Mean CSF-to-serum NOx level ratio values were significantly higher in patients with high severity on day-1 to day-4 (*P*-value < 0.001). The same trend was also evident in patients without vasospasm from day-1 to day-4 (*P*-value 0.001–0.016). Mean CSF-to-serum NOx ratio levels were higher in patients with poor outcomes but were not statistically significant. On review of the temporal changes in CSF-to-serum NOx ratio levels on day-1 to day-5 in patients based on severity, outcome and occurrence of vasospasm, the values steadily increased, before exponentially increasing on day-5 (Table 5). Comparison of CSF-to-serum NOx level ratios based on the occurrence of vasospasm and outcome from day-1 to day-4 showed statistically significant differences in patients with better outcomes, presence of vasospasm and high severity grading (*P*-values: 0.031, 0.043 and 0.034, respectively) (Table 6).

## Discussion

Patients with poor grades of IV and V aneurysmal subarachnoid hemorrhage based on the WFNS grading are treated conservatively, given their higher mortality and morbidity. This is especially the case for those with WFNS post-resuscitation-grade V, Fisher’s scale grade 3, non-middle cerebral artery (MCA) aneurysm, intracranial haemorrhage (ICH), intraventricular haemorrhage (IVH), brain herniation, symptomatic vasospasm, and delayed surgery, as all of the above are predictors of poor outcomes (20).

According to the general consensus in the animal model literature, there is an early decrease in NO in the period directly following SAH, presumably due to haemoglobin scavenging, consumption by neutrophils and interaction of NO with free radicals (12, 21–22). As demonstrated in the present study, a trend of steadily reducing NOx levels in CSF and serum was evident from days 1–4, likely due to the above-mentioned phenomena and possibly due to the continuous leakage of CSF from the external ventricular drainage. The subsequent small increase in NOx levels on day-5 can be explained by the early development of cerebral vasospasm, delayed cerebral ischaemia disease or correlated with an increase in intracranial pressure (15).

After SAH, the initial immediate peaks in CSF and serum NOx levels identified in our study following brain injury are probably due to the activity of endothelium NOx and neuronal NOx (23). Besides, in a study by Rejdak et al. (13), the immediate increase in NOx produced by neuronal NOx was effective in improving neurological outcomes in some models. After the initial peak in NOx, there is often a period of relative deficiency in NOx. This period of low NOx levels is associated with decreased cerebral blood flow (CBF).

Our study showed significant differences in NOx levels in CSF and serum in patients with SAH among the three subgroups. Serum NOx levels were statistically significantly higher in patients with high severity gradings and with radiological vasospasm. The elevation of NOx levels in serum occurs due to leakage from the CNS and can be caused by impairment to the BBB that occurs following SAH: the more severe the injuries, the more severe impairment of the BBB (18).

In our study, mean serum NOx level values in patients with outcome scores only showed significant results on day-1 and day-2; eventually, both subgroups achieved equal mean serum NOx level values. These differences in the results could be due to reduced production of NOx metabolites in serum; this has been hypothesised to result from the downregulation or inhibition of two of the various forms of NO synthase (NOS), endothelial NOS (eNOS) and neuronal NOS (nNOS) (24–25). Complicating our understanding of the role of NO in vasospasm is the hypothesis that inducible NOS (iNOS) may, in fact, be excessively active in vasospasm, with a resultant increase in free radical production and oxidative stress that can damage smooth muscle cells and lead to vasoconstriction (2, 26). Meanwhile, serum NOx levels should be interpreted with caution because their concentrations may be affected by many factors like nutritional nitrogen balance, bacterial metabolism in the GI tract and renal function (27).

Having found that, we correlated CSF and serum NOx levels with clinical outcome measures in SAH patients. No correlation was found between NOx and injury severity as assessed via WFNS grade. However, the mean values of CSF and serum NOx tend to be lower in patients with excellent outcomes compared to those with persistent moderate or severe disability or death, as compared to previous studies (21, 28).

In contrast to the above finding, a completely different result was obtained for NOx level in CSF among the three subgroups. There were statistically significant results in the mean values of CSF NOx levels among patients with severity and occurrence of vasospasm, where NOx levels were higher among those with high severity gradings and patients without vasospasm. This may be because of the opposing effect of NO reduction as a result of scavenging from oxyhemoglobin and the reduction of NOS activities in cerebral arteries versus an increase in NO production as a result of ischaemia due to vasospasm (29–31).

The ratio of CSF-to-serum NOx levels was selected for investigation mainly due to the various confounding factors that may influence the absolute value of serum NOx levels, nutritional nitrogen balance and bacterial metabolism in the intestine (22). Therefore, an extra cautious interpretation of the significance of serum NOx levels is required (27, 32). Our results were statistically significant for patients in terms of the CSF-to-serum NOx level ratio, which correlated with good outcomes and the presence of vasospasm and those with a high severity grading. We believe that this might be because more severe incidences of SAH result in a greater degree of BBB dysfunction, which further translates into an increased amount of NOx leaking from the CNS into the serum.

We compared the mean values and NOx levels ratios in CSF and serum between patients with and without vasospasm as measured via mean TCD velocities of the middle cerebral artery. The relevance of this was to determine whether the severity grading and outcome grading were associated with high levels of NOx. The increased level of NOx metabolites present in cerebral vasospasm could be explained as the result of an increase in inducible NOx, induced by the immunological response following SAH. Subsequent overproduction of NOx may cause free radical injury of cells of the vascular wall and provoke vasoconstriction. In contrast, the elevated nitrate/nitrite levels we observed could be an expression of pre-existing ischaemia, as an effect of cerebral vasospasm (2).

The decision whether or not to operate has always presented a dilemma for surgeons, especially for patients with poor WFNS grades (IV and V). Various authors have identified subgroups of poor WFNS-grade SAH patients with the potential to improve after aggressive treatment. Amongst selected patients, favourable outcomes can be achieved in as many as 47% of patients, with a mortality rate of 30%. Based on the currently available grading systems, none was able to provide a definite treatment regime, especially for poor-SAH grade patients (23, 28, 33).

Vasospasm is not the only factor contributing to poor clinical outcomes: a multitude of factors can contribute. For example, post-resuscitation WFNS V, non-MCA aneurysm, advanced age, IVH, ICH, brain herniation, delayed surgery and even systemic infection are predictors of poor clinical outcomes (20).

## Conclusion

The results suggested that elevated NOx levels in CSF and serum, and reductions in the NOx in CSF-to-serum ratio, were associated with severity, occurrence of vasospasm and clinical outcomes in aneurysmal SAH patients. It is recommended that the ratio of NOx in CSF-to-serum can potentially be used to predict which patients will have poor outcomes based on their GOS score and without vasospasm. Previously, vasospasm was a known predictor of severity and outcome in patients with SAH. The results indicate the possible role of NOx as a biomarker to assess severity and prognosis in patients with SAH. However, further research based on a larger sample size is required to study the CSF-to-NOx ratio as a potential marker in accurately predicting clinical outcomes in patients with SAH.

## Figures and Tables

**Table 1:** Demographic and clinical characteristics of patients

Characteristics	*n* (%)	mean (SD)
Age		58.2 (11.8)
Gender		
Male	12 (30.0)	
Female	28 (70.0)	
Ethnicity		
Malay	37 (92.5)	
Chinese	2 (5.0)	
Others	1 (2.5)	
Co-morbidities		
Diabetes mellitus	10 (25.0)	
Hypertension	28 (70.0)	
Others		
WFNS grading scale		
High (5–3)	26 (65.0)	
Low (1–2)	14 (35.0)	
Clinical parameters		
Anterior circulation aneurysm	38 (95.0)	
Posterior circulation aneurysm	2 (5.0)	
Vasospasm		
Present	18 (45.0)	
Absent	22 (55.0)	
Delayed cerebral ischaemia (DCI)		
Present	9 (22.5)	
Absent	31 (77.5)	
Severity grading		
High (13–3)	26 (65.0)	
Low (15–14)	14 (35.0)	
Outcome (GOS)		
Good	8 (20.0)	
GOS 5	3 (7.5)	
GOS 4	5 (12.5)	
Poor	32 (80.0)	
Gos 3	7 (21.9)	
Gos 2	13 (40.6)	
Gos 1	12 (37.5)	

**Table 2:** CSF, serum and ratio of CSF-to-serum NOx values for the study population

Day	CSF mean (SD)	Serum mean (SD)	Ratio mean (SD)
1	11.2 (25.9)	68.7 (85.9)	0.292 (0.207)
2	11.3 (20.4)	63.0 (77.7)	0.281 (0.157)
3	9.6 (16.9)	52.2 (65.2)	0.310 (0.148)
4	6.3 (6.8)	36.6 (44.1)	0.347 (0.179)
5	7.4 (10.3)	36.9 (38.4)	0.592 (1.127)
Overall	9.2 (2.3)	51.5 (15.0)	0.364 (0.130)

**Table 3:** Comparing mean (SD) CSF NOx levels on day1 till day 5 based on severity grading (WFNS), presence of vasospasm, presence of DCI and GOS at discharge and 6 months follow up

Mean NOx level by day (μm)	Conditions		*P*-value
	**Low severity grading (WFNS 1–2)**	**High severity grading (WFNS 3–5)**	

CSF NOx day 1	6.8 (2.3)	15.4 (35.1)	< 0.001
CSF NOx day 2	8.9 (7.4)	12.6 (24.9)	< 0.001
CSF NOx day 3	8.4 (9.8)	10.2 (19.9)	< 0.001
CSF NOx day 4	8.3 (11.0)	5.2 (2.4)	< 0.001
CSF NOx day 5	8.2 (14.0)	7.1 (7.8)	< 0.001

	**Without vasospasm**	**With vasospasm**	

CSF NOx day 1	13.2 (34.7)	8.7 (6.7)	< 0.001
CSF NOx day 2	12.5 (27.0)	9.8 (7.3)	< 0.001
CSF NOx day 3	10.4 (21.7)	8.6 (8.8)	< 0.001
CSF NOx day 4	6.5 (8.5)	6.0 (4.2)	< 0.001
CSF NOx day 5	7.6 (11.6)	7.3 (8.7)	< 0.001

	**DCI absent**	**DCI present**	

CSF NOx day 1	13.0 (32.5)	7.7 (5.0)	< 0.001
CSF NOx day 2	12.5 (25.3)	8.8 (7.1)	< 0.001
CSF NOx day 3	10.7 (14.1)	7.4 (5.9)	< 0.001
CSF NOx day 4	6.4 (8.1)	6.0 (4.7)	< 0.001
CSF NOx day 5	8.0 (11.7)	6.2 (5.1)	< 0.001

	**Poor outcome score (GOS 1–3)**	**Good outcome score (GOS 4–5)**	

CSF NOx day 1	12.9 (30.4)	6.8 (3.3)	0.099
CSF NOx day 2	12.6 (23.6)	7.8 (6.9)	0.054
CSF NOx day 3	11.0 (19.7)	5.8 (3.1)	0.030
CSF NOx day 4	6.4 (7.5)	6.0 (5.2)	0.006
CSF NOx day 5	8.3 (11.9)	5.1 (1.5)	0.011

**Table 4:** Comparing mean (SD) serum NOx level on day 1 till day 5 based on severity grading (WFNS), with/without vasospasm and GOS at discharge and 6 months follow up

Mean NOx level by day (μm)	Conditions		*P*-value
	**Low severity grading (WFNS 1–2)**	**High severity grading (WFNS 3–5)**	

Serum NOx day 1	50.7 (77.4)	78.4 (90.2)	0.800
Serum NOx day 2	49.3 (46.1)	70.3 (90.4)	0.882
Serum NOx day 3	41.4 (41.8)	58.0 (74.9)	0.292
Serum NOx day 4	26.7 (17.7)	42.0 (52.8)	0.005
Serum NOx day 5	28.3 (24.5)	41.5 (43.9)	0.003

	**Without vasospasm**	**With vasospasm**	

Serum NOx day 1	63.9 (85.7)	74.5 (88.4)	0.402
Serum NOx day 2	60.8 (90.0)	65.5 (62.0)	0.595
Serum NOx day 3	51.0 (73.2)	53.7 (55.8)	0.834
Serum NOx day 4	36.0 (47.7)	37.4 (40.5)	0.093
Serum NOx day 5	38.2 (42.4)	35.3 (34.1)	0.073

	**DCI absent**	**DCI present**	

Serum NOx day 1	66.0 (90.4)	72.7 (73.0)	0.032
Serum NOx day 2	61.9 (89.5)	64.3 (48.5)	0.029
Serum NOx day 3	52.4 (73.9)	52.0 (42.2)	0.051
Serum NOx day 4	35.8 (48.2)	38.2 (32.0)	0.234
Serum NOx day 5	37.4 (44.9)	36.1 (30.3)	0.246

	**Poor outcome score (GOS 1–3)**	**Good outcome score (GOS 4–5)**	

Serum NOx day 1	68.0 (95.2)	71.0 (58.5)	0.010
Serum NOx day 2	63.0 (89.3)	63.4 (35.1)	0.017
Serum NOx day 3	52.9 (75.0)	50.4 (28.3)	0.058
Serum NOx day 4	35.7 (49.8)	39.2 (25.2)	0.357
Serum NOx day 5	36.8 (41.6)	37.2 (30.3)	0.322

**Table 5:** Comparing mean (SD) ratio CSF to serum NOx level on day 1 till day 5 based on severity grading (WFNS), with/without vasospasm and Glasgow GOS at discharge and 6 months follow up

Mean NOx level by day (μm)	Conditions		*P*-value
	**Low Severity Grading (WFNS 1–2)**	**High Severity Grading (WFNS 3–5)**	

Ratio NOx day 1	0.271 (0.213)	0.302 (0.207)	< 0.001
Ratio NOx day 2	0.241 (0.126)	0.302 (0.170)	< 0.001
Ratio NOx day 3	0.286 (0.135)	0.324 (0.153)	< 0.001
Ratio NOx day 4	0.313 (0.141)	0.366 (0.197)	< 0.001
Ratio NOx day 5	0.710 (1.585)	0.529 (0.814)	0.773

	**Without vasospasm**	**With vasospasm**	

Ratio NOx day 1	0.345 (0.233)	0.226 (0.152)	0.003
Ratio NOx day 2	0.312 (0.178)	0.242 (0.121)	0.001
Ratio NOx day 3	0.330 (0.161)	0.286 (0.129)	0.005
Ratio NOx day 4	0.372 (0.186)	0.317 (0.172)	0.016
Ratio NOx day 5	0.617 (1.271)	0.561 (0.956)	0.829

	**DCI absent**	**DCI present**	

Ratio NOx day 1	0.334 (0.223)	0.217 (0.162)	< 0.001
Ratio NOx day 2	0.301 (0.167)	0.255 (0.141)	0.001
Ratio NOx day 3	0.327 (0.155)	0.280 (0.134)	0.003
Ratio NOx day 4	0.313 (0.192)	0.322 (0.148)	0.008
Ratio NOx day 5	0.554 (1.025)	0.711 (1.362)	0.089

	**Without vasospasm**	**With vasospasm**	

Ratio NOx day 1	0.345 (0.233)	0.226 (0.152)	0.003
Ratio NOx day 2	0.312 (0.178)	0.242 (0.121)	0.001
Ratio NOx day 3	0.330 (0.161)	0.286 (0.129)	0.005
Ratio NOx day 4	0.372 (0.186)	0.317 (0.172)	0.016
Ratio NOx day 5	0.617 (1.271)	0.561 (0.956)	0.829

	**DCI absent**	**DCI present**	

Ratio NOx day 1	0.334 (0.223)	0.217 (0.162)	< 0.001
Ratio NOx day 2	0.301 (0.167)	0.255 (0.141)	0.001
Ratio NOx day 3	0.327 (0.155)	0.280 (0.134)	0.003
Ratio NOx day 4	0.313 (0.192)	0.322 (0.148)	0.008

Ratio NOx day 5	0.554 (1.025)	0.711 (1.362)	0.089

	**Poor outcome score (GOS 3–5)**	**Good outcome score (GOS 1–2)**	

Ratio NOx day 1	0.323 (0.213)	0.208 (0.173)	0.742
Ratio NOx day 2	0.290 (0.157)	0.269 (0.162)	0.943
Ratio NOx day 3	0.324 (0.150)	0.275 (0.140)	0.654
Ratio NOx day 4	0.354 (0.198)	0.328 (0.124)	0.365
Ratio NOx day 5	0.491 (0.779)	0.860 (1.773)	0.089

**Table 6:** Association of CSF and serum towards ratio CSF to serum at baseline (day 1)

	Conditions		

Ratio	Low severity grading (WFNS 1–2)	High severity grading (WFNS 3–5)	*P*-value
CSF/serum	*n* (%)	*n* (%)	
< 0.3499	16 (61.5)	10 (71.4)	0.532
≥ 0.3500	10 (38.5)	4 (28.6)	
CSF/serum			
< 0.3999	18 (69.2)	11 (78.6)	0.528
≥ 0.4000	8 (30.8)	3 (21.4)	

	**Without vasospasm**	**With vasospasm**	

CSF/serum	*n* (%)	*n* (%)	
< 0.3499	13 (59.1)	13 (72.2)	0.386
≥ 0.3500	9 (40.9)	5 (27.8)	
CSF/serum			
< 0.3999	12 (42.9)	16 (57.1)	0.018
≥ 0.4000	10 (83.3)	2 (16.7)	

	**DCI absent**	**DCI present**	

CSF/serum	*n* (%)	*n* (%)	
< 0.3499	21 (67.7)	7 (77.7)	0.078
≥ 0.3500	10 (32.3)	2 (22.3)	
CSF/serum			
< 0.3999	25 (80.6)	8 (88.9)	0.029
≥ 0.4000	6 (23.3)	1 (11.1)	

	**Poor outcome score (GOS 3–5)**	**Good outcome score (GOS 1–2)**	

CSF/serum	*n* (%)	*n* (%)	
< 0.3499	13 (59.1)	9 (42.9)	0.036
≥ 0.3500	16 (88.9)	2 (11.1)	
CSF/serum			
< 0.3999	20 (69.0)	9 (81.8)	0.416
≥ 0.4000	9 (31.0)	2 (18.2)	

## References

[1] Al-Khindi T, Macdonald RL, Schweizer TA (2010). Cognitive and functional outcome after aneurysmal subarachnoid hemorrhage. Stroke.

[2] Woszczyk A, Deinsberger W, Boker DK (2003). Nitric oxide metabolites in cisternal CSF correlate with cerebral vasospasm in patients with a subarachnoid haemorrhage. Acta Neurochir (Wien).

[3] Nath S, Koziarz A, Badhiwala JH, Almenawer SA (2018). Predicting outcomes in aneurysmal subarachnoid haemorrhage. BMJ.

[4] (1988). Report of World Federation of Neurological Surgeons Committee on a universal subarachnoid hemorrhage grading scale. J Neurosurg.

[5] Hunt WE, Hess RM (1968). Surgical risk as related to time of intervention in the repair of intracranial aneurysms. J Neurosurg.

[6] Foreman PM, Chua MH, Harrigan MR, Fisher WS, Tubbs RS, Shoja MM (2017). External validation of the Practical Risk Chart for the prediction of delayed cerebral ischemia following aneurysmal subarachnoid hemorrhage. J Neurosurg.

[7] Jaja BN, Cusimano MD, Etminan N, Hanggi D, Hasan D, Ilodigwe D (2013). Clinical prediction models for aneurysmal subarachnoid hemorrhage: a systematic review. Neurocrit Care.

[8] Chao CC, Hu S, Molitor TW, Shaskan EG, Peterson PK (1992). Activated microglia mediate neuronal cell injury via a nitric oxide mechanism. J Immunol.

[9] Moncada S, Palmer R, Higgs E (1991). Nitric oxide: physiology, pathophysiology, and pharmacology. Am Soc Pharm Exp Ther.

[10] Gross SS, Wolin M (1995). Nitric oxide: pathophysiology mechanism. Annu Rev Physiol.

[11] Sadamitsu D, Kuroda Y, Nagamitsu T, Tsuruta R, Inoue T, Ueda T (2001). Cerebrospinal fluid and plasma concentrations of nitric oxide metabolites in postoperative patients with subarachnoid hemorrhage. Crit Care Med.

[12] Vellimana AK, Milner E, Azad TD, Harries MD, Zhou ML, Gidday JM (2011). Endothelial nitric oxide synthase mediates endogenous protection against subarachnoid hemorrhage-induced cerebral vasospasm. Stroke.

[13] Rejdak K, Petzold A, Sharpe MA, Kay AD, Kerr M, Keir G (2004). Cerebrospinal fluid nitrite/nitrate correlated with oxyhemoglobin and outcome in patients with subarachnoid hemorrhage. J Neurol Sci.

[14] Clark RS, Kochanek PM, Obrist WD, Wong HR, Billiar TR, Wisniewski SR (1996). Cerebrospinal fluid and plasma nitrite and nitrate concentrations after head injury in humans. Crit Care Med.

[15] Kandasamy R, Kanti Pal H, Swamy M, Abdullah J (2013). Cerebrospinal fluid nitric oxide metabolite levels as a biomarker in severe traumatic brain injury. Int J Neurosci.

[16] Bratasz A, Kuter I, Konior R, Gościński I, Łukiewicz S (2004). Nitric oxide as a prognostic marker for neurological diseases. Antioxid Redox Signal.

[17] Ng WH, Moochhala S, Yeo TT, Ong PL, Ng PY (2001). Nitric oxide and subarachnoid hemorrhage: elevated level in cerebrospinal fluid and their implications. Neurosurgery.

[18] Germano A, d’Avella D, Cicciarello R, Hayes RL, Tomasello F (1992). Blood-brain barrier permeability changes after experimental subarachnoid hemorrhage. Neurosurgery.

[19] Rangel-Castilla L, Robertson CS (2007). Nitric oxide metabolism after traumatic brain injury. Intensive Care Med.

[20] Zhao B, Tan X, Zhao Y, Cao Y, Wu J, Zhong M (2016). Variation in patient characteristics and outcomes between early and delayed surgery in poor-grade aneurysmal subarachnoid hemorrhage. Neurosurgery.

[21] Pluta RM, Thompson BG, Dawson TM, Snyder SH, Boock RJ, Oldfield EH (1996). Loss of nitric oxide synthase immunoreactivity in cerebral vasospasm. J Neurosurg.

[22] Sehba FA, Schwartz AY, Chereshnev I, Bederson JB (2000). Acute decrease in cerebral nitric oxide levels after subarachnoid hemorrhage. J Cereb Blood Flow Metab.

[23] Starke RM, Komotar RJ, Otten ML, Schmidt JM, Fernandez LD, Rincon F (2009). Predicting long-term outcome in poor grade aneurysmal subarachnoid haemorrhage patients utilising the Glasgow Coma Scale. J Clin Neurosci.

[24] Pluta RM (2005). Delayed cerebral vasospasm and nitric oxide: review, new hypothesis, and proposed treatment. Pharmacol Ther.

[25] Sabri M, Ai J, Knight B, Tariq A, Jeon H, Shang X (2011). Uncoupling of endothelial nitric oxide synthase after experimental subarachnoid hemorrhage. J Cereb Blood Flow Metab.

[26] Crowley RW, Medel R, Kassell NF, Dumont AS (2008). New insights into the causes and therapy of cerebral vasospasm following subarachnoid hemorrhage. Drug Discov Today.

[27] Dondorp AM, Planche T, de Bel EE, Angus BJ, Chotivanich KT, Silamut K (1998). Nitric oxides in plasma, urine, and cerebrospinal fluid in patients with severe falciparum malaria. Am J Trop Med Hyg.

[28] Takagi K, Tamura A, Nakagomi T, Nakayama H, Gotoh O, Kawai K (1999). How should a subarachnoid hemorrhage grading scale be determined? a combinatorial approach based solely on the Glasgow Coma Scale. J Neurosurg.

[29] Kurzelewski M, Czarnowska E, Beresewicz A (2005). Superoxide- and nitric oxide-derived species mediate endothelial dysfunction, endothelial glycocalyx disruption, and enhanced neutrophil adhesion in the post-ischemic guinea-pig heart. J Physiol Pharmacol.

[30] Rejdak K, Petzold A, Stelmasiak Z, Giovannoni G (2008). Cerebrospinal fluid brain specific proteins in relation to nitric oxide metabolites during relapse of multiple sclerosis. Mult Scler.

[31] Siuta M, Zuckerman SL, Mocco J (2013). Nitric oxide in cerebral vasospasm: theories, measurement, and treatment. Neurol Res Int.

[32] Ghasemi A, Zahedi Asl S, Mehrabi Y, Saadat N, Azizi F (2008). Serum nitric oxide metabolite levels in a general healthy population: relation to sex and age. Life Sci.

[33] Hutchinson PJ, Power DM, Tripathi P, Kirkpatrick PJ (2000). Outcome from poor grade aneurysmal subarachnoid haemorrhage--which poor grade subarachnoid haemorrhage patients benefit from aneurysm clipping?. Br J Neurosurg.

